# Using a “Kickoff” to build implementation partner teams and action plans for active implementation of a quality improvement project

**DOI:** 10.3389/frhs.2025.1580653

**Published:** 2025-06-10

**Authors:** Sean A. Baird, Teresa M. Damush, Nicholas A. Rattray, Lauren S. Penney, Edward J. Miech, Barbara J. Homoya, Jared Ferguson, Laura J. Myers, Dawn M. Bravata

**Affiliations:** ^1^ Department of Veterans Affairs (VA), Health Services Research (HSR) Expanding Expertise Through E-health Network Development (EXTEND) Quality Enhancement Research Initiative (QUERI) Center, Indianapolis, IN, United States; ^2^ Department of Veterans Affairs (VA) Health Systems Research (HSR) Center for Health Information and Communication (CHIC), Richard L. Roudebush VA Medical Center, Indianapolis, IN, United States; ^3^ Department of Internal Medicine, Indiana University School of Medicine, Indianapolis, IN, United States; ^4^ Health Services Research (HSR), Regenstrief Institute, Indianapolis, IN, United States; ^5^ VA HSR&D Elizabeth Dole Center of Excellence for Veteran and Caregiver Research, South Texas Veterans Health Care System, San Antonio, TX, United States; ^6^ School of Medicine, University of Texas Health San Antonio, San Antonio, TX, United States; ^7^ Department of Neurology, Indiana University School of Medicine, Indianapolis, IN, United States

**Keywords:** kickoff, implementation strategies, quality improvement, action plan, team

## Abstract

**Introduction:**

A strategy for transitioning implementation successfully from pre-implementation to active implementation is to hold a team “kickoff.” The objectives of this manuscript are: (1) to present the frameworks that guided the development of the Protocol-guided Rapid Evaluation of Veterans Experiencing New Transient neurological symptoms (PREVENT) kickoff strategy, (2) describe design elements of the kickoff and how they contribute to achieving PREVENT kickoff aims; forming teams, developing an action plan, and launching active implementation (3) examine the perceived usefulness of those kickoff design elements toward achieving kickoff aims.

**Methods:**

PREVENT was a stepped-wedge trial to improve the quality of Transient Ischemic Attack (TIA) care at six Veterans Affairs (VA) medical centers. The PREVENT kickoff was designed from multiple frameworks: theory of change principles for process improvement; Consolidated Framework for Implementation Research (CFIR); social learning models; and systems redesign. Data collected included pre-kickoff planning documents and post-kickoff debriefs from the PREVENT national team, Audience Response System (ARS) data, post-kickoff site participant evaluations and semi-structured interviews.

**Results:**

Site team participants reflected positively on the framework driven, interactive and interpersonal design elements, team building, and action plan exercises, and found them useful for a successful project launch. In-person and hybrid set-up of the kickoff, interactive elements, and team formation activities emphasized the quality problem, and motivated site implementation providers to plan for stroke/TIA care improvement.

**Conclusions:**

Implementation team kickoffs during pre-implementation are a useful approach to inform and engage local clinical teams and to form plans for practice changes to improve clinical care.

**Clinical Trial Registration:**

clinicaltrials.gov, identifier NCT02769338.

## Introduction

Evidence-based programs typically move from pre-implementation to active implementation when key implementers are oriented, trained, and equipped to begin implementation. One strategy for launching implementation is to convene a team “kickoff.” (1–4) Kickoffs, or similar events which launch projects, appear across several fields including: implementation research (1, 5), community based or multi-stakeholder research (6), project management (7), and systems engineering [e.g., Rapid Process Improvement Workshops (RPIW), Lean Six Sigma, Systems Redesign] (1, 8, 9). Kickoff events commonly include (1): interactive design elements (1, 6), introduction and orientation to relevant content (1, 6), tools and products to be used during active implementation (1, 6), team-building activities (1, 6), and strategies for planning or charter creation (1, 6, 7). However, few empirical studies have examined how kickoffs are created and tailored, especially for health care settings, and few offer any perspective of the teams' clinical providers and staff on its usefulness for planning quality improvement (5).

Our team had previously used kickoff meetings using Systems Redesign elements of collaborative learning where concurrent teams across sites kickoff together to bridge pre-implementation to implementation (3). However, we noticed gaps in team development and subsequent practice changes using this approach. Therefore, we designed a team kickoff building upon our prior work and using theoretical frameworks to promote teamwork and practice changes, while tailoring the delivery to each team based on baseline team development, organization, relationships and experiences.

This manuscript focuses on the development and evaluation of the PREVENT kickoff strategy (1). The specific aims are: (1) to present the development of the PREVENT kickoff design guided by frameworks, (2) describe the kickoff strategy design elements, and (3) evaluate the usefulness of those kickoff elements in forming site teams, informing the development of site action plans, and moving those teams into active implementation with the “buy-in” to implement PREVENT.

## Materials and methods

### The PREVENT study

PREVENT was a stepped wedge trial with three waves; two sites per wave (*n* = 6 facilities overall); each facility had a one-year active implementation period that was started by a kickoff (1). PREVENT was designed to facilitate clinical teams' evidence-based practice to improve the quality of care for patients with transient ischemic attacks (TIA) at Veteran Affairs (VA) facilities across the U.S (2). PREVENT focused on seven processes of TIA and stroke care that have been associated with improved patient outcomes. The PREVENT intervention included five components: a quality of care reporting system, clinical programs, professional education, electronic health record tools, and quality improvement support (1, 3). The PREVENT implementation timeline included four main elements: pre-implementation phases with facility baseline interviews, and early facilitation between national QI implementation team and prospective site partner participants; a site kickoff event that gathered site participants to form a local implementation team, develop an action plan, and launch active implementation; the 1-year active implementation phase involving facilitation, learning collaboratives, and access to data reporting and other resources; and a 6-month sustainment period with reduced facilitation and collaborative opportunities.

The PREVENT national QI implementation team (national team) was multi-disciplinary with members from general internal medicine, implementation and social sciences, nursing, quality management, and data science backgrounds. Their role was to evaluate and facilitate with site partner participants to implement PREVENT and improving seven processes of care via site visits, kickoffs, regular check-ins, and collaborative calls throughout the PREVENT evaluation (1). Based on prior stroke-related QI projects (3), the national team recognized the need for an implementation strategy that would effectively form local site teams delivering TIA and stroke care, equipped with the knowledge and action plan to launch active implementation at their site.

A full-day, in-person team kickoff meeting was conducted at each of the six PREVENT sites to gather participants from diverse clinical settings and transition sites from pre-implementation to active implementation. The local site Champion (the leader for site implementation of PREVENT) identified site participants from across disciplines and services (e.g., neurology, pharmacy, emergency medicine, radiologists, hospitalists, and other primary care staff) who were invited to the kickoff. Site partner participants (site team) of PREVENT were responsible for attending the kickoff, developing their sites action plan for implementing PREVENT, participating in collaborative calls, and reflecting on facility data about seven processes of TIA care. The PREVENT study was approved by the Indiana University institutional review board and the Richard L. Roudebush Department of Veterans Affairs (VA) research and development committee (protocol #1511914238).

### Conceptual frameworks guiding kickoff design

Four conceptual frameworks informed the PREVENT kickoff design seeking to enhance team formation and activate implementation: Theory of Change (10–12), Social Learning Model (13), Systems Redesign (SR) (9, 14), and Consolidated Framework for Implementation Research (CFIR) (1, 15). The frameworks were operationalized via agenda items and kickoff design elements (features that were incorporated into the kickoff design but are not necessarily agenda items).

**Figure 1:**
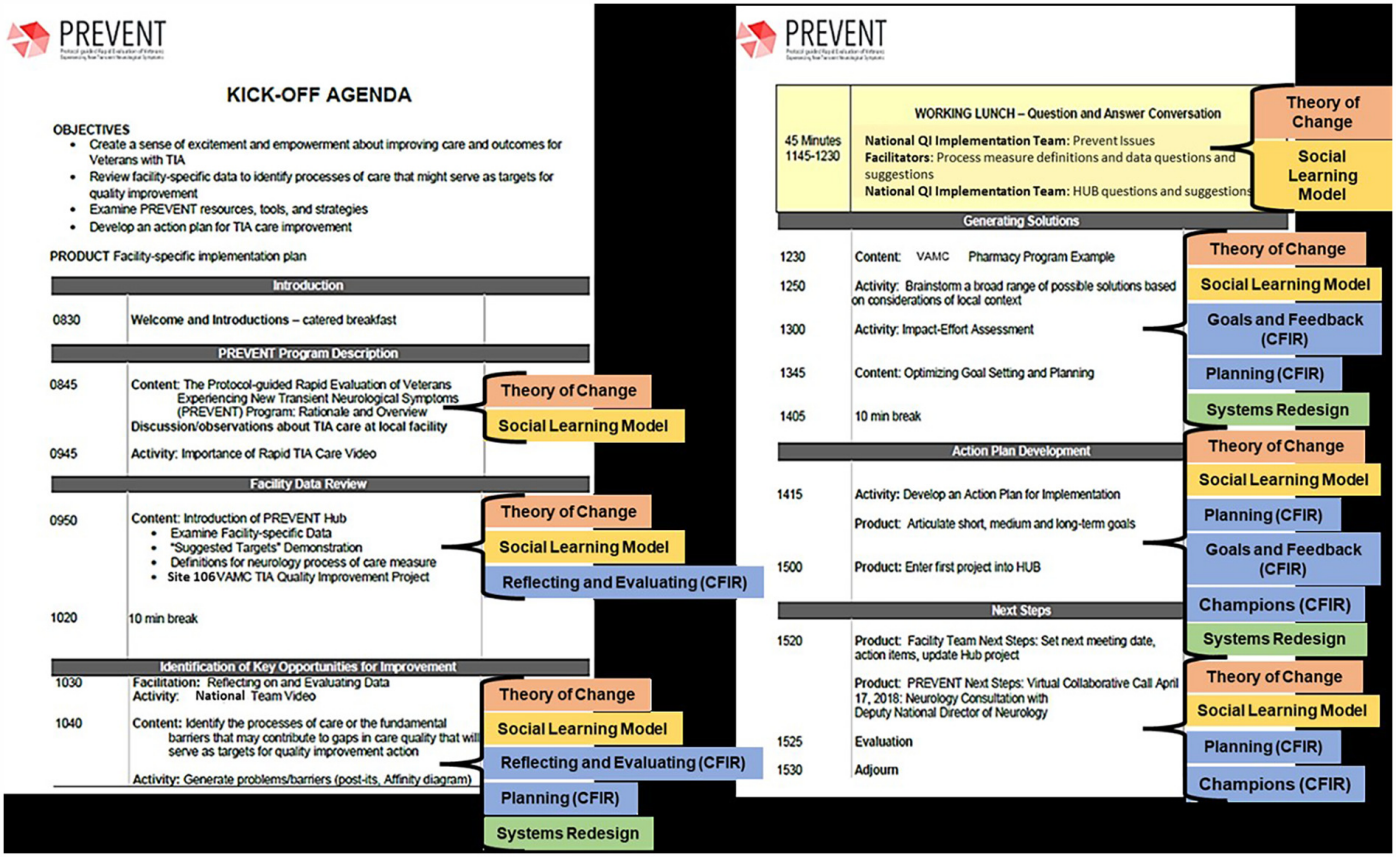
Example kickoff agenda overlayed with identified frameworks by agenda segment.

The main aims of the kickoff were to launch active implementation, facilitate a diverse group of clinical providers to form a team to engage in other collaborative PREVENT implementation strategies (4, 16), and develop a site partner-determined action plan to improve TIA care at their facility. Table 1 lists kickoff agenda items and design elements along with their associated conceptual framework that item operationalized and intended application. Figure 1 features a site kickoff agenda overlayed with operationalized frameworks.

**Table 1:** Kickoff design elements, frameworks and applied framework elements, and mechanisms of change.

Kickoff design element/ agenda item	Operationalized framework(s) and framework elements applied to Kickoff agenda	Mechanisms of change
In-person, hybrid format	• Theory of change: alliance strengthening, convincing change agents • CFIR: relative priority, mission alignment (between facility and QI)	• Intended to convey importance of implementing PREVENT • Facilitate engagement of site team formation and conducting activities to support action plan development
Meals, food, and breaks	• Theory of change: alliance strengthening, capacity building • Social learning theory: positive reinforcements	• Drawing on previous experiences attending long, sometimes full day, education seminars and project launches with few breaks, food, or any consideration for participants • The national team designed the kickoff to have consideration for the site team, and build positive and productive collaboration between site and national teams; answer questions
Content: PREVENT and discussion about facility TIA care	• Theory of change: Capacity building • Social learning model: Observational learning • CFIR: access to knowledge and information	• Presentation on PREVENT provided an opportunity for the site participants to arrive, meet each other, meet the national team, and learn about PREVENT
Importance of rapid TIA care video	• Theory of change: Convincing change agents • Social learning model: Observational learning (of TIA cases), consequences of cases	• TIA care videos offer another media and addition to educating site team participants to further convince site implementers the importance of improving seven processes of TIA care
Audience response system (ARS)	• Social learning model: stimulus response learning (Remote ARS responses and real time feedback) • CFIR: reflecting and evaluating	• ARS allowed site team participants to engage and provide feedback to the national QI implementation team
Introduction of PREVENT hub Reflecting on and evaluating data	• Theory of change: capacity building • Providing measures and outcomes • Social learning model: performance feedback • CFIR: reflecting and evaluating, available resources • Systems redesign: current state and quality goals	• The hub is an interactive electronic data dashboard and resource site that sites could use to monitor their performance data, implementation progress, and share and view resources • Sites were presented with the hub and defined seven processes of care with facility data, building their capacity to use the hub
National team video	• Theory of change: alliance strengthening • Social learning model: socially modeling (viewing VA clinical team model team behavior to improve QI), reinforce team behaviors (showing that team's success (outcomes))	• The national QI implementation used created video demonstrations using examples from previous work to socially model team formation and practice changes • The video featured ad a successful VA stroke team discussing how they work together to improve stroke care processes
Identifying processes of care or fundamental barriers Problems and barriers affinity diagram with post-its	• Theory of change: understanding context, alliance strengthening, convincing change agents • Social learning model: Planning practice • CFIR: tension for change, planning, reflecting and evaluating • Systems redesign: identifying Kapowies (barriers or challenges) and solutions	• Prior to the first kickoff, the national team tested several different planning activities to facilitate the site team building their site's action plan • The national team used the affinity diagram activity from SR using post-its • Intent was for the site team to start working together to identify barriers and build their action plan
Other VAMC Pharmacy program	• Theory of change: capacity building • Social learning model: observational learning from protocol and ability to locally adapt • Systems redesign: example of QI	• The national team presented a case example of a Pharmacy program that modeled improving TIA care at a facility by improving pharmacy processes of care
Brainstorming solutions Impact-effort assessment	• Theory of change: understanding context, Alliance strengthening, • Social Learning Model: Practice planning • CFIR: Planning, Goals & feedback • Systems Redesign: Impact-Effort assessment to apply solutions	• The national team used an SR activity to facilitate further team planning and developing goals to integrate into the site's action plans
Optimizing goal setting and planning	• Theory of change: backwards mapping to bridge the objectives with the outcomes • Social learning model: planning practice changes, motivation to change based on impact-effort • CFIR: planning, goals & feedback specifying the context in which goals and plans will occur • Systems redesign: applying the impact-effort assessment	• Using the impact-effort assessment and solutions, the site team identify goals for active implementation and how to move forward • Sites are nearly directing the kickoff at this point and actively planning their site's active implementation
Develop the action plan	• Theory of change: alliance strengthening, capacity building • Social learning model: practice plan and motivation to change • CFIR: planning, goals & feedback • Systems redesign: plan, study, act, do cycles	• Site teams formalize their action plan, the main objective and product of the kickoff • The site team participants agree as a team on the action plan, formalize and have it stored on the interactive electronic data dashboard and resource site
Evaluation	• Theory of change: alliance strengthening • CFIR: planning, feedback	• Site teams complete an evaluation for the national team to use for future kickoffs

#### Theory of change principles of process improvement

Theory of change identifies and utilizes mechanisms to facilitate change in a system or setting through explicit declaration of an end goal and process (4). Theory of change principles relevant to process change, implementation, and PREVENT's program needs included understanding context and preconditions, providing measures and outcomes, capacity building of team members, alliance strengthening among all parties, and convincing change agents to facilitate implementation (17). The kickoff itself was the initial operationalization of mechanisms of change in the local site processes and system to implement and sustain improvements for TIA and stroke care. Theory of change is applied with almost every agenda item from interactive team activities and planning to strengthen alliances, to conducting in-person meetings and opportunities to review and reflect on facility data to convince change agents (See Table 1).

#### Social learning model

The PREVENT team adopted a social learning model approach to education (13, 14) which emphasizes observing and modeling; where new information is presented with opportunities to practice and apply information being modeled for observers. The social learning model provided an approach for engaging site staff to build their own plan for implementing PREVENT. Specifically, team examples from previous work were presented to socially model team formation and practices changes including a video from a successful VA stroke team discussing how they work together to improve stroke care processes (See Table 1).

#### Systems redesign

Systems Redesign (SR) focuses on making systematic changes to improve patient care from an effectiveness, efficiency, and quality standpoint (9, 14, 18). SR is a widespread process improvement program within VHA, and the implementation team had used SR approaches successfully in other QI projects (19, 20). PREVENT sought to improve processes of TIA care within the individual facility's systems of stroke care. Although SR projects include multi-day events, we selected applicable SR processes and activities that could be included in a single-day kickoff. The specific SR activities were selected based on experience with their use from other projects, pilot-testing, and alignment with kickoff and PREVENT program aims (18). SR activities such as identifying barriers to ideal processes, brainstorming solutions, and mapping solutions onto an impact-effort matrix were used in the kickoff to guide site team members to collaboratively develop their site-specific action plan (See Table 1) (7, 9, 21).

#### Consolidated framework for implementation research (CFIR)

The Consolidated Framework for Implementation Research (CFIR) provides typology for implementation mechanisms within a health services setting; constructs and mechanisms commonly used in successful implementation (22). We used CFIR both to guide implementation development and as an evaluating framework for all of PREVENT implementation. Results from previous stroke teams practice changes and PREVENT baseline interview data directed the national team to focus on operationalizing four key CFIR constructs associated with successful implementation: Planning, Goals and Feedback, Reflecting and Evaluating, and Supporting Champions (1, 23). During the PREVENT kickoff each key construct along with other identifiable constructs (e.g., mission alignment, relative priority, available resources, and others) were demonstrated with applied examples, for instance reviewing facility data operationalizes Reflecting and Evaluating and creating the action plan operationalizes Planning (see Table 1).

### Kickoff agenda

All design elements and agenda items were used to meet the objectives of the kickoff. To convey importance of PREVENT, the PREVENT national team conducted the kickoff in-person with some remote staff using videoconferencing in a single full working day. Due to the constraints on clinicians schedules the kickoff was limited to a single day, and time had to be used wisely to get necessary work done to have the site team complete an action plan.

The kickoffs introduced the PREVENT protocol and major components for improving TIA care (1). Site team members were guided through an interactive electronic data dashboard and resource site that site teams could use to monitor their performance data, implementation progress, and access resources. After, site staff and providers were presented with the rationale for focusing on the seven TIA processes of care, external facilitation methods, tools and resources, and quality metrics to measure TIA care. The site team then used SR activities (e.g., identifying barriers to ideal processes, brainstorming solutions, and mapping solutions onto an impact-effort matrix) to discuss local barriers, identify actionable tasks and needs to improve TIA care at their site and incorporate into their action plan. In line with social learning model, SR strategies allowed site staff and providers to engage in the learning process, and moving from learning and observing to planning and goal setting (also a CFIR construct) and preparing for implementing PREVENT with the added layer of simultaneously forming at team (see Table 1; Figure 1).

### Site staff and providers comforts and conveniences

To make for an experientially positive kickoff, comforts and conveniences for the site staff and providers, and activities to encourage site staff and providers' engagement were included in the kickoff design. For example, the kickoffs were conducted in-person at each VA facility at a time that was most convenient for personnel at that site. Being in person helped convey the importance of PREVENT, but also to build an interpersonal connection between the national team and the site team. We also explicitly recognized that the facility providers and staff were the local experts most equipped to identify the barriers, and with the most knowledge to implement PREVENT at their site. The national team attempted to avoid a hierarchical approach to the site team to present and demonstrate where needed, but mostly allow the site team to direct preparing for active implementation.

Interpersonal communication conveyed the national team's commitment to engage site teams as full partners. Engagement with site teams required substantial investments in: communication with site staff and providers, planning and coordination between the national team and the site facility, and logistics. Site leadership (i.e., chief-of-staff) were encouraged to attend the kickoff to convey the importance of implementing the PREVENT program and facilitate “buy-in” among site team members. Certificates and tokens of appreciation were given to the kickoff partners to further mark the significance of the kickoff, emphasize a partnership to implement PREVENT, and deputize the newly formed team to implement PREVENT.

## Evaluation

### Data collection

To evaluate the PREVENT kickoffs, we collected data from planning and debriefing documents from the national team and data collected from 51 site team kickoff participants from across the six sites. There were five main sources of data: (1) Pre-kickoff planning materials, (2) post-kickoff debriefing sessions, (3) during kickoff participant feedback from the Audience Response System, (4) post-kickoff evaluations, and (5) semi-structured interviews collected during active implementation.

Pre-kickoff planning materials consisted of memos and meeting notes from the national team documents outlining ideas, agenda items, site information related to planning, creating, and preparing kickoffs at sites. Post-kickoff debriefing sessions (*n* = 6) were conducted for each site by the national team, which were recorded and transcribed. These sessions were opportunities for the PREVENT national team to describe what went well, what needed improvement, early impressions of a site team and note participant dynamics, and to discuss next steps for each site.

An all-digital, real-time feedback method, an Audience Response System (ARS), was used during the kickoff to engage staff and providers and elicit their perspectives. ARS questions had multiple choice responses designed to rate kickoff elements. Kickoff participants responded to questions anonymously using remote control “clickers.” Responses from ARS participants were downloaded for analysis.

Kickoff evaluations (*n* = 32) consisted of five open-ended questions designed for kickoff participants to provide feedback on what they appreciated and gained from the kickoff, and describe next steps for active implementation at their site (see Supplementary Appendix A). The evaluations were administered at the end of the kickoff meeting. Evaluation responses were anonymous and handwritten, but later transposed into an Excel (Microsoft) spreadsheet.

Semi-structured, audio recorded, interviews were conducted in-person with site staff and providers (*n* = 32) at 6 months after a site's kickoff, during active implementation. Interview questions were asked about the impact of the team kickoffs on local active implementation. Interviews were recorded and transcribed for thematic coding analysis.

### Mixed methods analysis

Analysis of the data aimed to: (1) map the frameworks of the kickoffs and PREVENT to design of the kickoff and its agenda items, (2) understand the kickoffs development and usefulness from two separate perspectives; the national team and site team (3) identify if and how the agenda items and incorporated frameworks helped meet the kickoff main objectives, i.e., forming teams and developing action plans.

Kickoff planning materials were analyzed by converting documents into an NVivo file for analysis. Pre-kickoff planning documents went through an open coding process (24) to map overarching, pre-identified frameworks and some emergent sub-frameworks and constructs. Planning material coding was then triangulated with coded post-kickoff debriefs and six-month semi-structured interviews.

Post-kickoff debrief transcripts were open coded in NVivo to identify content related to kickoff planning, adaptations, and theories or frameworks, as well as descriptions of the perceived reception of the kickoff (e.g., relevance for site personnel, critical or positive feedback).

Semi-structured interviews were performed by the national team. Interview questions were focused on kickoff implementation and site staff's experiences. Interviews were transcribed and coded independently by two members of the evaluation/research team merged with partner coders, and consensus coded for agreement on use of the codebook. The codebook was based on implementation domains, principally from CFIR, and project-specific codes related to PREVENT (e.g., kickoff). Additionally, a round of categorical coding was applied to the six-month interviews, where participant signifiers and quantifiable codes, codes such as clinical role, Likert question scores, provider years in practice, etc. were applied to interviews to provide further cross-analysis with other codebooks (24).

ARS responses were tabulated to show the totals on kickoff elements site personnel found most useful across sites (SB, BH, TMD). Post-kickoff evaluation responses were collected and compared across sites and reviewed for common responses about site staff members' perceptions of the kickoff (SB). Perceptions and preferences from evaluations were compared across tables with ARS response tables. SB reviewed and conducted open coding of post-kickoff debrief transcripts for kickoff data related to underlying theories, planning and adaptation elements for kickoffs, and site perceptions of the kickoff. SB analyzed kickoff code reports from 6-month interview transcripts, conducted additional categorical coding and comparing coded 6-month interviews to kickoff coded excerpts from pre-kickoff planning material and post-kickoff debriefs. Transcript coding and analysis of 6-month, post-kickoff debriefs, and pre-kickoff planning documents was performed using NVivo12 software for data coding and analyses (25).

## Results

The results show how each respective kickoff design element and framework contributed toward each of the main aims of the kickoffs: developing an action plan, forming collaborative teams and partnerships, and launching active implementation. Kickoff design elements often operationalized more than one framework, each agenda item and design element worked toward achieving multiple aims at a time. Additionally, site team data shows which design elements were more useful, appreciated, or helped generate “buy-in” for participants to begin active implementation.

### Participants

There was a total of 51 site team kickoff participants across six sites. There were 32 unique site staff and providers who participated in anonymous post-kickoff evaluations (*n* = 32) and 6-month interviews (*n* = 32); an average of 5 (range 3–9) site personnel from each VA site (*n* = 6) completed evaluations and six-month interviews. ARS responses were aggregated across site attendees; responses to ARS were received from participants who were present and willing to respond when questions were presented during kickoffs.

### Planning and action plan

Several activities went into planning for improving the quality of acute TIA care at each local site. *Planning* was an operationalized CFIR construct, and opportunities to plan implementation of PREVENT at each site were intentionally woven throughout the kickoff agenda (see Table 1; Figure 1). A main objective of the kickoff was the development of a site-specific action plan with inputs from the newly formed multi-disciplinary team. SR activities like diagraming barriers, facilitators, and goals on an impact-effort matrix, and making an action plan were all a part of *Planning* at the site. The way the kickoffs provided the opportunity to plan as a multi-disciplinary team was appreciated by participating personnel. From ARS responses, site participants were asked about their top preferred kickoff elements, and half of the top responses were related to team planning and developing the action plan; “Team Brainstorming solutions”—18.68%, “Team Developing Plan Do Study Act/Action Plan”—14.02% (see Table 2).

**Table 2:** Site teams identified most helpful design elements from the kickoff.

Kickoff element	QI teams enrolled in PREVENT						
	101 (*n* = 8)^a^	102 (*n* = 8)^a^	103 (*n* = 11)^a^	104 (*n* = 10)^a^	105 (*n* = 7)^a^	106 (*n* = 7)^a^	Overall sample mean %
Reviewing facility performance data on the hub	27.27%	23.53%	13%	15.15%	12%	100%	31.83%
Team brainstorming solutions	27.27%	17.65%	15%	15.15%	12%	25%	18.68%
Meeting with other staff members from my facility	9.09%	23.53%	15%	15.15%	18%	–	16.15%
Team developing “Plan Do Study Act” (PDSA)/Action Plan	18.18%	11.76%	13%	15.15%	12%	–	14.02%
Post-its of local problems team activity	9.09%	0.00%	13%	6.06%	18%	–	9.23%
Handouts	0.00%	17.65%	9%	9.09%	6%	–	8.35%
Illustrative videos	0.00%	0.00%	4%	9.09%	6%	–	8.03%
Pharmacy program quality improvement example	9.09%	5.88%	9%	9.09%	6%	–	7.81%
Team Impact-effort assessment	0.00%	0.00%	9%	6.06%	12%	–	5.41%

Note: To rate the usefulness of the team's day long Kickoff, respondents were instructed to “Please identify the parts of the Kickoff were most useful (all that apply) (Multiple Choice).” Teams 101–105 used an automated response system (ARS). Due to technical difficulties, Team 106 listed useful elements on an open-ended response sheet where participants had to recall the elements.

^a^ Sample size is equal to the total site team participants who were at the respective site kickoff and available to potentially respond to the ARS question but is not the exact number of participant responses for a given question.

### Team forming

The six sites had varied associations and teams among site staff prior to their kickoffs. Some had established and collaborative neurology teams before the PREVENT kickoff. Other sites had staff meeting each other for the first time at the kickoff. The national team commented on debriefs about the positive team dynamics either being evident right at the beginning of kickoffs or being formed throughout the kickoff. Team formation suffered when facility personnel at some sites were unable to attend the kickoff meeting, or when facility staff had to come in and out of the full day meeting to attend to other clinical duties. From ARS data, “Meeting with other staff members from my facility,”—16.15% was one of the top four preferred kickoff elements (see Table 2). In Table 3, site staff and providers expressed that the kickoff was an excellent opportunity for the different site personnel to meet with each other, “[get] everybody on the same page,” and get different perspectives from different providers for planning. In fact, some sites expressed that they wished they had had more relevant site staff and providers who were not present at the kickoff.

**Table 3:** Site participant perspectives on PREVENT kickoff design elements.

Site	Kickoff design elements	Quotes
101	• Team forming	…Things that I think about, [NEUROLOGIST] might not think about and vice versa… It's helpful for everyone to be their kind of as a team that has different views on what should be done…—101_6m_1
	• Planning • Developing goals	I feel like that was the 1st time we've, we really kind of drilled down on a good… starting plan; kind of an outline of what we wanted to do, and what we wanted to accomplish… I feel like we started that [at the Kickoff]. So I think it played a very large role in… how we decided we wanted to proceed.—101_6m_1
	• Team forming • “Buy-in” on PREVENT • Reflecting and Evaluating	It was very clear to us what we needed to do… being able to get everybody on the same understanding, sort of that shared knowledge of how the without fail could reduce the likelihood of stroke… I think it was actually even surprising to us as emergency physicians that it was more effective than we realized, and we knew that our colleagues would buy into that… you're doing this because you should be doing it, but rather that there was actual the likelihood of reducing strokes.—101_6m_6
	• In-person emphasized importance	It was probably more effective to have the meeting… The fact that you guys came on site emphasizes or heightens the importance of it.—101_6m_6
102	• Education • Planning • Developing goals	I came out of it feeling that I knew what the issue is. I knew what the problem is. I know what they're trying to address. I know what the goal is, and I have information sources so that I'm able to do it—102_6m_3
	• Team forming	I had a little time to talk to different people and really… one of the biggest things that I see is that I think that it really helped to come up with more of a team… I have more of a working relationship with neurology now, which I didn't have before. - 102_6m_3
	• Team forming (desire for more to be included)	I thought that a barrier was [attendance]. Some key players weren't there. I think that internal medicine wasn't there, or the hospitalists weren't there. I thought that they should have been involved… I thought that that was one kind of limitation. In our smaller groups though, we ended up meeting with them anyway. So, I mean, it didn't have that much of a negative effect on the overall project, but I just thought that it would be good to have all of the key players at the first kickoff meeting.—102_6m_4
103	• “Buy-in” on PREVENT • In-person emphasized importance	I think it was nice that you guys came in person and then the other team members participated remotely… I don't think I would have done anything different… I admit I was a little… skeptical at first about how it was all going to work, but I thought it was fantastic. - 103m_6m_6
	• Team forming • In-person emphasized importance	I think that the kickoff was critical. Getting the right people there. Blocking off that chunk of time… Just to show that hey, we're committed to this, and let's get this launch right. Doing a webinar or that type of thing without getting the whole group together would have been tough. We'd all have been sitting in the offices doing three other things at the same time. - 103_6m_1
104	• Planning • “Buy-in” on PREVENT	I honestly think that it goes back to the kickoff meeting. I think that the kickoff meeting was the main source of motivation; having ideas, discussing them, coming up with plans. Being that involved and engaged, I think is what might have made me follow through with them. Because I mean after that, I felt like I had all of these great ideas, and I wanted to actually implement them.—104_6m_4
	• Developing goals • Action plan • SR impact effort assessment	I think that for me it was our action plan and goals and writing everything out was really nice on a board… I think that we did… a high risk/high reward… Like a homerun kind of thing. I think that that really helped a lot.—104_6m_4
105	• Education • Team forming • Planning (desire for more)	I definitely appreciated more education information about TIA care and the PREVENT research and protocols that we have established. It was really nice to sit with some people from different disciplines and find out about not only what they do but their ideas or passion for wanting to improve care for TIA patients in [THIS VAMC]. I think that the only thing that I would have changed would be getting more time for us on that day to think about what we had to do.—105_6m_2
106	• In-person emphasized importance • Virtual physical layout of the room	I think it was outstanding. I think it went very well. I loved the idea of having extended table with [virtual attendees]. I think it worked great. I have actually mentioned it to other groups that they should do that.—106_6m_1 Note: The physical room was set up with a table that extended towards a screen with virtual attendees at table, giving the look of a continuous table with in-person and virtual participants at the same table
	• Education (laying out process)	I think they laid the [processes of PREVENT] out pretty clearly.—106_6m_2

Note: The ID placed after each quote indicates the site, date of the phase of interview during active implementation, and the participant.

### “Buy-in” among facility personnel

The team kickoff structure was consistent across sites with adjustments made based upon lessons learned from post-kickoff debriefs and individual site needs. Minor changes were made to improve the flow of the meeting and allocating time for agenda items, engaging specific site personnel, setting up at venues within different VAMCs, and highlighting opportunities for improvement based on baseline quality performance. Interviewed providers stated that kickoffs and design considerations helped build “buy-in” among site personnel to improve the seven TIA processes of care. “Buy-in” is the state in which the national team was able to emphasize the importance of implementing PREVENT and improving the seven TIA processes of care, and the site team were convinced and committed to implementing PREVENT at their site. Being shown how effective PREVENT and improving the seven TIA processes of care could be for improving care for Veterans with stroke or TIA helped kickoff participants, as they stated, “buy into” PREVENT (see Table 3). From interview data, kickoff elements that helped convince skeptical site staff and providers included the in-person meeting, being presented with site data (*Reflecting and Evaluating*), considerations for site staff and providers comforts and other intangibles, and the physical set up of the kickoff venues (see Table 3). Furthermore, ARS data's top response for preferred kickoff elements was “Reviewing facility performance data on the hub”—31.83%, demonstrating how demonstrating how reflecting and evaluating activities and presenting site's performance data was for participants (See Table 2).

## Discussion

The PREVENT national team drew on several frameworks related to process or system change and their own experiences with previous program launch events to develop the PREVENT site kickoff strategy, demonstrating an intentional comprehensive design. Moreover, the PREVENT national team conducted kickoff participant evaluations to actively improve iterations of the kickoff for successive PREVENT sites; and to evaluate the recipients' perceptions of the intentionally designed kickoff. The results demonstrate the effectiveness in the kickoffs: every site developed a facility-specific action plan, and the events promoted team formation and buy-in to implement PREVENT.

Using an intentionally designed kickoff as an implementation planning meeting is aligned with the literature on implementation program launches (7, 26). Within the field of implementation science, several distinct implementation strategies align with kickoff design elements, such as providing education, training on a specific aspect of the evidence based practice, creating new local teams, or having a local setting action plan for moving to active implementation (26). For PREVENT, the kickoffs helped with forming the site teams and set up the teams for later collaborative strategies like collaborative calls and facilitator engagement that contributed to PREVENT's overall success (16). The site action plans from the kickoffs helped some site clinical champions to press their site team to implement all their original goals before active implementation phase ended (27).

The Intervention for Stroke Improvement using Redesign Engineering (INSPIRE) featured similar implementation strategies as PREVENT, primarily with a program initiating “face-to-face collaborative training” for VHA site stroke teams (20). INSPIRE held a multi-day, learning collaborative with external facilitation bringing together six site teams at a centralized location e.g., developing Plan-Do-Study-Act cycles, experiential learning, identifying operational barriers (20). The PREVENT kickoff built upon lessons learned and experiences from INSPIRE including the need to tailor the kickoff to each site team and then build the collaborative from across these individual site teams. Although literature describes the INSPIRE training strategies and elements (19), no literature details the frameworks or rationale for including specific components, nor the perception of participants.

Mendel et al. 2011 described a kickoff utilized as a “community engagement” strategy for a community-partnered participatory research QI program delivering depression interventions, Community Partners in Care (CPIC) (6). The kickoff discussed was a “three-quarter day” and featured modes of engagement appropriate for the community setting to educate and get community members to engage with provided depression interventions (6). Although some participants were affiliated with healthcare settings, the kickoff and CPIC were aimed to serve and meet participants outside of a healthcare system (6). The CPIC study described guiding frameworks for how the kickoff was designed including agenda items, logistics, and evaluation of kickoff participants' perspectives (6). The CPIC team articulated similar concerns as the PREVENT investigators in terms of addressing hierarchical dynamics between facilitators and participants.

Within Veteran Health Administration (VHA), Pittman et al. 2021 had discussed using Rapid Process Improvement Workshops (RPIWs) at multiple VHA sites to implement eScreening (8). The eScreening RPIWs were three-day events to plan and launch implementation for multiple VHA sites concurrently (8). Pittman et al. offered details on their agenda, and involved evaluations that described the perspective of participants on the RPIW, its elements, and impact on implementation (8). The RPIW, while determined to be useful, was also not utilized by all sites.

PREVENT was a QI program operating in a structured healthcare system that took place at VHA facilities across the US. PREVENT modeled the kickoff to align with VHA standards and practices for process improvement, while contending with varying local settings and multiple types of providers. The PREVENT kickoff strategy offered an approach for team implementation within diverse healthcare settings with action plans for successful implementation in each setting.

### Limitations

The kickoff was a key implementation strategy for launching active implementation for PREVENT at each participating site during pre-implementation. However, there was no control group; therefore, we are unable to evaluate how effective the kickoffs were in comparison to an alternative approach. The team kickoffs were a component of a larger set of implementation strategies previously reported (1, 2, 16, 21, 27).

## Conclusion

The PREVENT kickoff implementation strategy succeeded at launching active implementation at each site and contributed to individual sites' implementation success by bringing together diverse sets of site providers and staff to form collaborative teams with the knowledge and buy-in to implement PREVENT. The kickoffs guided site teams to build site-specific action plans, prepared to begin active implementation.

## Data Availability

The datasets presented in this article are not readily available because these data must remain on Department of Veterans Affairs servers; investigators interested in working with these data are encouraged to contact the corresponding author. Requests to access the datasets should be directed to sean.baird@va.gov.
