# Supplementary material for: Using a “Kickoff” to build implementation partner teams and action plans for active implementation of a quality improvement project
Source: Front Health Serv. 2025 Jun 10;5:1580653. doi: 10.3389/frhs.2025.1580653 (PMC12185449; doi:10.3389/frhs.2025.1580653)
Supplement: Supplementary file 1 [file Datasheet1.pdf]

PREVENT

Protocol-guided Rapid Evaluation of Veterans Experiencing New Transient Neurological Symptoms

KICK-OFF EVALUATION

Identify one part of the Kick-Off that surprised you:

Our goal was for the Kick-Off to be enjoyable and useful. Please identify one new tool, strategy, or piece of information that you found to be particularly valuable:

In the next week, what one new thing will you do to improve TIA care?

What major challenges do you see in terms of implementing PREVENT at this facility in the near future?

Please list any suggestions that you for us to improve future PREVENT Kick-Off sessions:
